# Criterion distances and environmental correlates of active commuting to school in children

**DOI:** 10.1186/1479-5868-8-88

**Published:** 2011-08-10

**Authors:** Sara D'Haese, Femke De Meester, Ilse De Bourdeaudhuij, Benedicte Deforche, Greet Cardon

**Affiliations:** 1 Faculty of Medicine and Health Sciences, Department of Movement and Sports Sciences, Ghent University, Watersportlaan 2, 9000 Ghent, Belgium; 2 Department of Human Biometrics and Biomechanics, Vrije Universiteit Brussel, Brussels, Belgium

## Abstract

**Background:**

Active commuting to school can contribute to daily physical activity levels in children. Insight into the determinants of active commuting is needed, to promote such behavior in children living within a feasible commuting distance from school. This study determined feasible distances for walking and cycling to school (criterion distances) in 11- to 12-year-old Belgian children. For children living within these criterion distances from school, the correlation between parental perceptions of the environment, the number of motorized vehicles per family and the commuting mode (active/passive) to school was investigated.

**Methods:**

Parents (n = 696) were contacted through 44 randomly selected classes of the final year (sixth grade) in elementary schools in East- and West-Flanders. Parental environmental perceptions were obtained using the parent version of Neighborhood Environment Walkability Scale for Youth (NEWS-Y). Information about active commuting to school was obtained using a self-reported questionnaire for parents. Distances from the children's home to school were objectively measured with Routenet online route planner. Criterion distances were set at the distance in which at least 85% of the active commuters lived. After the determination of these criterion distances, multilevel analyses were conducted to determine correlates of active commuting to school within these distances.

**Results:**

Almost sixty percent (59.3%) of the total sample commuted actively to school. Criterion distances were set at 1.5 kilometers for walking and 3.0 kilometers for cycling. In the range of 2.01 - 2.50 kilometers household distance from school, the number of passive commuters exceeded the number of active commuters. For children who were living less than 3.0 kilometers away from school, only perceived accessibility by the parents was positively associated with active commuting to school. Within the group of active commuters, a longer distance to school was associated with more cycling to school compared to walking to school.

**Conclusions:**

Household distance from school is an important correlate of transport mode to school in children. Interventions to promote active commuting in 11-12 year olds should be focusing on children who are living within the criterion distance of 3.0 kilometers from school by improving the accessibility en route from children's home to school.

## Background

Being physically active can help to reduce the prevalence of obesity in children [1], is associated with a decrease in cardiovascular risk factors [2] and may reduce the risk of osteoporosis at older age [3]. At 11-12 years of age however, physical activity levels rapidly decline [4-6]. As a high level of physical activity in 9- to 18-year-olds predicts a high level of adult physical activity [7], it is important to promote physical activity during childhood.

Active commuting to school can contribute to achieving the recommended physical activity levels in elementary schoolchildren [8-10] of at least 60 minutes of moderate to vigorous physical activity (MVPA) per day [11]. In a study of Cooper et al., children who walked to school were significantly more physically active than those who travelled by car [8]. Cycling to school was associated with higher overall physical activity levels, only in boys [8]. Sirard et al. showed in the USA, that regularly active commuting children from elementary school from the fifth grade (mean age 10.3 ± 0.6 yr), were approximately 24 minutes more engaged in MVPA per day [10]. These findings emphasize the importance of promoting active commuting in children from elementary school to enhance physical activity levels in children. However, to promote active commuting in elementary school, it is necessary to gain insight into correlates of active commuting behavior in schoolchildren.

Recently, ecological models got increasing attention. The focus of ecological models is on the determination of built and natural environmental causes of behavior [12]. Besides the physical environment, interpersonal and cultural factors also influence behavior according to the ecological model [13]. It is hypothesized that environmental factors can influence behaviors as well directly as indirectly [14]. These theories suggest a profound investigation of the neighborhood environment in order to create suitable interventions.

According to the review of Panter et al., the physical environment is one of the four main domains that can influence active travel behavior [15]. Other affecting factors are individual factors (e.g. physical ability, parental characteristics, motivation,..), external factors (e.g. weather, cost of travel and government policy), and main moderators (age, gender and distance to destination) [15]. As the physical environment is changeable, and a change of the environment can have positive influences for the whole community, insight into this domain may be indispensable, with an eye on creating appropriate interventions to encourage active commuting in schoolchildren.

According to the review of Panter et al. [15], one of the most important and consistent predictors of active commuting to school, is the household distance from school. Household distance from school is negatively associated with active commuting to school. Australian children were more likely to actively commute to school if their route was < 800 meters [16]. Moreover, Merom et al. [17] showed, that the number of Australian schoolchildren that did not actively commute to school doubled when distance increased from 750 m to 1500 m. As household distance from school is the most important predictor of active commuting to school, investigating other predictors for children living within a feasible distance from school for active commuting is of interest. Therefore, criterion distances for walking and cycling; which represent feasible distances for active commuting to school in elementary school children should be determined. Van Dyck et al. (2010), determined criterion distances for Belgian older adolescents (17-18 years) for both cycling (8.0 kilometers) and walking (2.0 kilometers) [18]. As older adolescents might have a greater independent mobility compared to children, and independent mobility may differ between children of different ages, it is necessary to determine more age-specific criterion distances for Belgian children. Passive commuters, who are living within these criterion distances, should be the focus of interventions to promote active commuting to school.

The environmental correlates of active commuting to school in children within these criterion distances, need to be revealed. Several perceived environmental factors have been identified as predictors of children's travel behavior [15,19,20]. Studies in Australia [21] and the USA [22] highlighted that parental concern about safety was associated with less walking and cycling to school. Parental perceptions of no traffic lights or crossings for their child to use, good connectivity en route to school and having to cross busy roads to get to school were all negatively associated with walking or cycling to school in Australia [16]. Results from a national survey in the USA suggested that having sidewalks is an important feature to promote active commuting to school in children [23]. Alton et al. [24], found in the UK that child perceptions of parental concern about heavy traffic and unsafe streets were associated with more walking in general. In Portugal, a positive association was found between street connectivity and walking to school [25]. Panter et al. [26] found in the UK a moderating effect for distance, whereby attitudes were more important for short distances and safety concerns for long distances. The possession of more than one car per household seemed not to be associated with active commuting to school in one Australian study [17] whereas in another Australian study, lesser car ownership was associated with more walking to school [27]. Furthermore, studies rarely investigated the correlates for walking and cycling separately. However, de Vries et al. showed that environmental correlates of walking and cycling in children, differ by purpose and commuting mode [28]. Therefore, it is necessary to investigate environmental correlates separately for walking and cycling, and for the different purposes such as active commuting to school, active commuting during leisure time and recreational walking or cycling [28].

According to the review of Panter et al. [15] most studies that revealed environmental correlates of active commuting were conducted in the USA and in Australia. In this review, 13 out of 24 studies were conducted in the USA, 7 studies were conducted in Australia and only 4 studies were conducted in Europe (Norway, Portugal, the Netherlands and the UK) [15]. It is likely that, in these countries, other predictors are responsible for active travel behavior to school; due to a different design and use of urban areas in which motor vehicle use is strongly existing, when compared to Flanders in Belgium. Flanders is the Dutch-speaking part of Belgium. The mild sea climate, the flat landscape and the dense network of cycle tracks (12.000 kilometers cycle tracks) make from Flanders a cycle-friendly region in which the prevalence of walking and cycling in general is much higher compared to other countries [29]. Moreover, more than 80% of the Flemish households own at least one bike [30]. Children in Belgium are not obliged to wear bicycle helmets. Mostly, bikes are stored at common places at school and theft is not a problem at elementary schools.

In conclusion, there is lack of age-specific criterion distances and, consequently, lack of European evidence concerning environmental correlates of active commuting to school in children living within their age-specific criterion distances.

Consequently, the aim of this study was to determine criterion distances for walking and cycling to school in Flemish 11- to 12-year-old children. After the determination of these criterion distances, multidimensional correlates of transport mode choice to school were examined for children living within the criterion distance from school.

## Methods

### Procedure

All data of the present study were obtained through the parents. Parental reports of active commuting to school were included in this study; as they are considered to be more reliable compared to children's data at that age [31]. Parental perception of the neighborhood was used instead of children's data as the framework by Panter et al. defined the parents as the most important decision makers for the choice of travel method to school in children [15].

The parents were reached through the schools of their child. In total, 148 schools were randomly selected from all elementary schools in East- and West-Flanders in Belgium and contacted by phone. From these schools, 44 principals agreed to let the sixth grade classes of their school participate (response rate schools = 42,9%) and gave written informed consent. The rather low response rate of 42.9% of the schools was comparable to other prior studies, based on questionnaires for pupils and parents, and is due to the fact that schools have many obligations and are consequently not very keen on spending time on research activities.

From each school, only one randomly selected class was included in the study to guarantee sufficient diversity in the dataset. The number of pupils per class varied from 6 to 23, and children were mainly 11-12 years old.

Through these 44 classes, 996 parents (one parent per child) could be reached. The parents of 696 children gave informed consent and were involved in the study (response rate parents = 69,9%). Children took the questionnaires from school to home and parents completed the questionnaire at home.

The 70% response rate of the parents was high and as 49.3% of the parents included in the study obtained a college or university diploma, this is a slightly higher percentage compared to 41.2% of the 25 to 29 year old people in 2007 [32]. The mean age of the parents' children was 11.2 ± 0.5 years of which 52.0% were boys. Data were collected between October 2009 and May 2010. The Ethics Committee of the Ghent University Hospital approved the study.

### Measures

#### Sociodemographic information

Parents were asked to fill in their own age, gender, and their level of education and their partner's level of education. Educational attainment was used as a measure for SES, as educational attainment is easy to measure and is fairly stable beyond early adulthood, and higher levels of education are usually associated with better jobs, housing, neighborhoods, working conditions and higher incomes [33]. Families were classified as high SES-families if the educational level of at least one parent was of a college or a university level; families were classified as low SES families if none of both parents reached a college or a university education level. Parents were also asked to fill in the number of motorized vehicles in their family.

#### Active commuting

The part of the questionnaire about active commuting was based on the validated Flemish Physical Activity Questionnaire (FPAQ) [34]. The questionnaire included the question: 'How does your child usually go to school?' There were three response categories: on foot, by bike, or with motorized transport (by car, train or bus). The time it took to go from home to school for their child, was also asked in the questionnaire. Furthermore, the parents were asked to indicate on which days their children usually came home during lunchbreak. Based on this information, the number of minutes that children were weekly engaged in active commuting to school was calculated. Parents also filled in their household address. Routenet online route planner (http://www.routenet.be) was used to objectively determine the distance of the shortest route from each child's home to school.

#### Environmental perceptions

The parent version of the 'Neighborhood Environment Walkability Survey for Youth' (NEWS-Y) in Dutch was used to determine environmental perceptions of the neighborhood. Internal consistency for all subscales and test-retest reliability of NEWS-Y for parents of 5-11 year old children was found to be acceptable with an intra class correlation range from 0.56 for street connectivity to 0.87 for crime safety [35].

The NEWS-Y determines the perceptions of residential density, the accessibility and diversity of land use mix, street connectivity, walk- and cycle infrastructure, aesthetics of the neighborhood and crime- and traffic safety. All determinants were calculated following the NEWS-Y scoring guidelines [36] with a higher score, denoting better conditions for active commuting. An outline of the questions is presented in table 1. All questions were rated on a five point scale and recoded were necessary. Response options are represented in table 1.

**Table 1:** Outline of the NEWS-Y parent version

Nr	Questions about the neighborhood	n	Parental mean
**1**	**Residential density *(None/A few/Half/Most/All the residences)***	**632**	**79.30**

*1a*	*How common are separate or stand alone one family homes?*	667	3.09 ± 1.23
*1b*	*How common are connected townhouses or row houses?*	671	2.92 ± 1.09
*1c*	*How common are apartment or condo buildings?*	642	1.68 ± 0.79
**2**	**Accessibility *(Strongly disagree/Somewhat disagree/Sometimes I agree; Sometimes I disagree/Somewhat agree/Strongly agree)***	**692**	**3.47 ± 1.14**

*2a*	*From our home, it is easy to walk to school*.	688	3.25 ± 1.68
*2b*	*There are many places where my child can walk to, alone or with someone else*.	691	3.27 ± 1.42
*2c*	*It is easy to walk from one place to another (there is no motorway, railway or river)*.	687	3.73 ± 1.27
*2d*	*It is easy to walk to a play garden or a park*.	686	3.62 ± 1.40
**3**	**Land use mix *(1-5 min/6-10 min/11-20 min/21-30 min/> 30 min)***	**684**	**3.34 ± 1.02**

	*How long should it take to walk to....*		
*3a*	*convenience/small grocery store?*	646	3.59 ± 1.22
*3b*	*supermarket?*	668	3.07 ± 1.34
*3c*	*bakery?*	677	3.82 ± 1.16
*3d*	*butcher's?*	669	3.54 ± 1.23
*3e*	*newspaper stand?*	670	3.49 ± 1.27
*3f*	*bank?*	641	2.97 ± 1.34
*3g*	*library?*	666	2.79 ± 1.32
**4**	**Street connectivity *(Strongly disagree/Somewhat disagree/Sometimes I agree; Sometimes I disagree/Somewhat agree/Strongly agree)***	**689**	**3.40 ± 0.91**

*4a*	*The streets have many cul-de-sacs*.	686	3.56 ± 1.28
*4b*	*There are many intersections*.	687	3.24 ± 1.15
**5**	**Walking/Cycling facilities *(Strongly disagree/Somewhat disagree/Sometimes I agree; Sometimes I disagree/Somewhat agree/Strongly agree)***	**692**	**2.71 ± 0.88**

*5a*	*There are sidewalks on most of the streets*.	691	3.36 ± 1.37
*5b*	*There are bikeways on most of the streets*.	688	2.53 ± 1.21
*5c*	*Bikeways are separated from the road/traffic by parked cars*.	688	2.06 ± 1.11
*5d*	*There are bicycle sheds (at supermarkets, schools, bus stops...)*.	686	2.91 ± 1.21
**6**	**Neighbourhood aesthetics *(Strongly disagree/Somewhat disagree/Sometimes I agree; Sometimes I disagree/somewhat agree/strongly agree)***	**694**	**3.27 ± 0.88**

*6a*	*There are many trees along the streets*.	694	3.09 ± 1.211
*6b*	*There is a beautiful scenery. (e.g. a beautiful landscape or view)*	694	3.43 ± 1.25
*6c*	*There are many buildings/homes that are nice to look at*.	693	3.29 ± 0.98
**7**	**Traffic safety *(Strongly disagree/Somewhat disagree/Sometimes I agree; Sometimes I disagree/Somewhat agree/Strongly agree)***	**692**	**2.85 ± 0.68**

*7a*	*Walking is dangerous because of the traffic*.	690	3.23 ± 1.04
*7b*	*Cycling is dangerous because of the traffic*.	691	2.84 ± 1.04
*7c*	*Cars usually drive slowly*.	690	2.45 ± 0.99
*7d*	*Our streets have good lightning at night*.	691	3.31 ± 0.99
*7e*	*There are crosswalks and signals to help walkers cross busy streets*.	689	3.04 ± 1.12
*7f*	*It is safe to play on the streets*.	691	2.24 ± 1.278
**8**	**Crime safety *(Strongly disagree/Somewhat disagree/Sometimes I agree; Sometimes I disagree/Somewhat agree/Strongly agree)***	**693**	**3.46 ± 0.74**

*8a*	*There is a low crime rate*.	686	3.79 ± 0.94
*8b*	*It is necessary to be afraid of strangers when I am/my child is walking down the street alone*.	666	3.33 ± 1.03
*8c*	*It is necessary to be afraid of when I am/my child is alone in a playground or a park*.	661	3.25 ± 1.06
*8d*	*My bike is safe when I lock it*.	669	3.44 ± 1.02

Residential density was calculated by following formula: score on question 1a + 12*score on question 1b + 25*score on question 1c. All the other subscales were scored by taking the mean of the different question scores on a 5 point scale.

Following the NEWS-Y rating scale [36], residential density was calculated by the following formula: score on question 1a + 12*score on question 1b + 25*score on question 1c. All the other subscales were scored by taking the mean of the different question scores. A measure for walkability was obtained by using following formula: walkability z-score = z-score residential density + 2*z-score connectivity + z-score land use mix [37].

### Data analysis

SPSS 15.0 was used to describe the characteristics of the sample.

Two-level bivariate regression analyses were conducted using MLwiN version 2.20. A two-level hierarchical model (school-pupil) was used to take into account clustering of children in schools. Two-level regressions investigated the relationship between the children's transport mode choice to school (active/passive commuting: dummy variable), and household distance from school.

Two-level logistic regressions investigated the relationship between the children's transport mode choice to school (active/passive commuting: dummy variable), and family SES (high/low: dummy variable) and gender (boy/girl: dummy variable).

Criterion distances for walking, cycling and passive commuting to school, were determined by examining cumulative percentages of children commuting to school by bike, on foot and in a passive way, per covered distance. Criterion distances were set at the distance in which at least 85% of the active commuters lived [18]. These distances were supposed to be feasible distances for children to actively commute to school.

After determination of these criterion distances, correlates of active commuting to school for children living within these feasible distances were determined. Therefore, multivariate regression analyses were conducted using MLwiN version 2.20. Two-level logistic regressions investigated the multivariate relationship between the children's transport mode choice to school (active/passive commuting: dummy variable), and parental neighborhood perceptions and number of motorized vehicles per family in the first model. In a second model, the relationship between active transport mode (on foot/by bike: dummy variable) and the independent variables was examined. Household distance from school was included as controlling variable within the second model. The multilevel analyses were both controlled for gender and SES of the parents. For each independent variable, odds ratio and confidence interval were given in table 2.

**Table 2:** Logistic multi-level analyses of sociodemographic and environmental correlates of transportation mode to school

Dependent variable:	ACTIVE (= 1) OR PASSIVE (= 0) COMMUTING TO SCHOOL n = 503			ACTIVE COMMUTING BY BIKE (= 1) OR ON FOOT (= 0) n = 369		
*Predictor:*	β ± SD	OR	95% CI	β ± SD	OR	95% CI
*SES (ref: low SES)*	0.272 ± 0.271	1,31	0.77 - 2.23	-0.346 ± 0.312	0,71	0.38 - 1.30
*Gender (ref: male)*	-0.092 ± 0.325	0,91	0.48 - 1.72	-0.131 ± 0.367	0,88	0.43 - 1.80
*Number of motorized vehicles*	-0.327 ± 1.049	0,72	0.09 - 5.64	-0.460 ± 1.097	0,63	0.07 - 5.42
*Walkability*	0.047 ± 0.057	1,05	0.94 - 1.17	-0.026 ± 0.066	0,97	0.86 - 1.11
*Accessibility*	**0.607 ± 0.146**	**1,83**	1.38 - 2.44*	-0.272 ± 0.200	0,76	0.51 - 1.13
*Walk/cycle facilities*	0.250 ± 0.168	1,28	0.92 - 1.78	-0.261 ± 0,182	0,77	0.53 - 1.11
*Neighborhood aesthetics*	-0.110 ± 0.166	0,9	0.65 - 1.24	0.005 ± 0.185	1,01	0.70 - 1.44
*Safety from traffic*	0.045 ± 0.239	1,05	0.65 - 1.67	0.287 ± 0.281	1,33	0.77 - 2.31
*Safety from crime*	0.013 ± 0.195	1,01	0.69 - 1.48	2,331 ± 0,226	1,26	0.81 - 1.96
*Household distance from school*				**1.980 ± 0.531**	**7,24**	2.56 - 20.51*

SE = standard error, 95% CI = 95% confidence interval, OR = odds ratio

* p < 0.05

Before executing the multivariate analyses, multicollinearity among independent variables was tested by performing pearsons' correlations. We used the value of r > 0.4 as an indication of collinearity [38]. None of the variables was excluded as there were no correlations > 0.4 found. Two independent variables, household distance from school and number of motorized vehicles, were initially skewed (skewness > 0.7). Therefore, logarithmic transformations (log_10_) were made to improve normality of these two independent variables [39]. All continuous variables were mean centered before they were inserted into the models [40].

For all analyses, P-values ≤ 0.05 were considered as significant.

## Results

### Descriptive characteristics

Almost sixty percent (59.3%) of the total sample commuted actively to school (38.1% by bike and 21.2% on foot). Of the active commuters, 54.5% were boys and 45.5% were girls. In the girls' subsample, 55.5% commuted to school in an active way whereas more than sixty percent (63.0%) of the boys commuted actively to school. These differences were not significant (OR = 1.324; CI = 0.974 - 1.802). According to SES, there were no differences found in commuting mode to school in children (OR = 0.914, CI = 0.652 - 1.280).

Children lived on average 2.96 ± 3.97 kilometers away from school (range: 0.05 - 33.50 kilometers). Passive commuters lived further away from school (4.70 ± 4.67 kilometers) compared to active commuters (1.73 ± 2.83 kilometers) (p < 0.001). The mean duration of an active trip (by bike or on foot) to school was 9.4 ± 6.0 minutes. A biking trip took 9.7 ± 6.1 minutes and a trip by foot took 8.8 ± 5.8 minutes on average. Children, who commuted actively to school, were engaged in active commuting for 111.4 ± 69.1 minutes weekly.

### Determination of criterion distances

**Figure 1:**
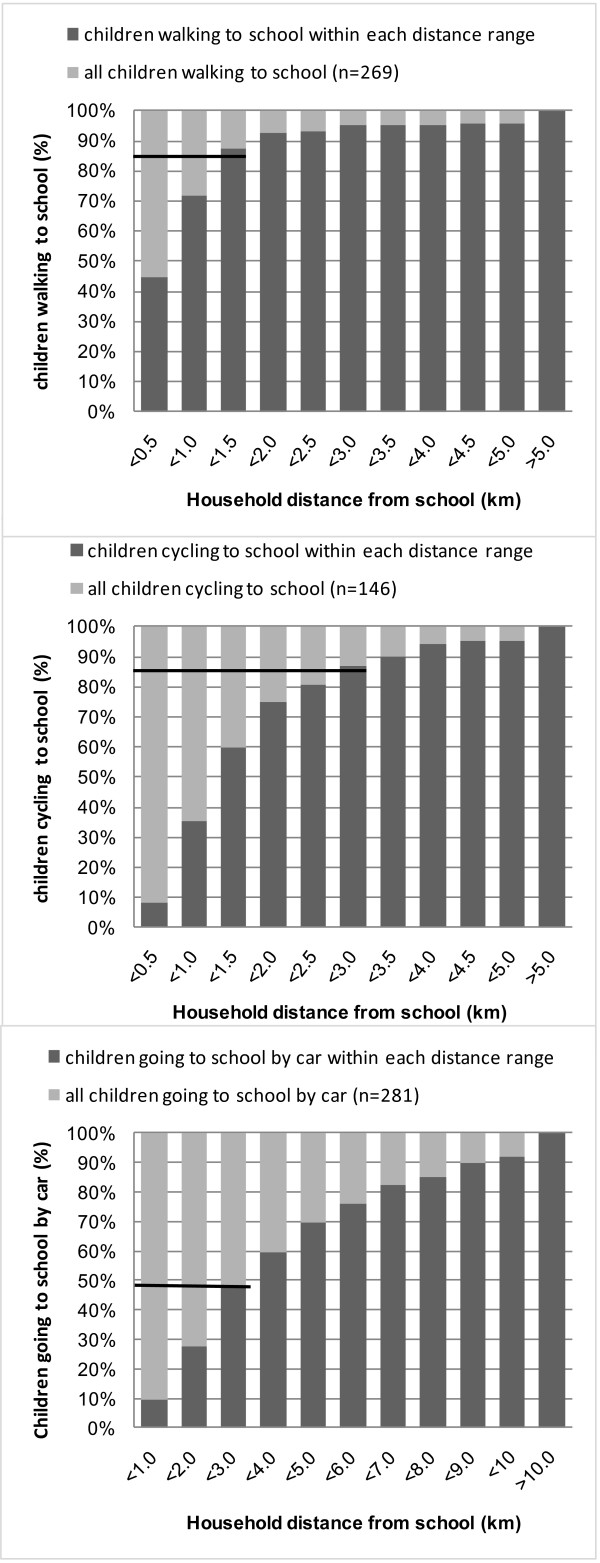
**Cumulative percentage of covered distance ranges per transportation mode**.

Figure 1 shows that 86.4% of the children who walk to school lived within 1.5 kilometers from school. Of all children who cycled to school, 86.8% lived less than 3.0 kilometers away from school. Therefore, criterion distances were set at 1.5 kilometers for walking and 3.0 kilometers for cycling to school in Belgian 11-12 year old children. Of the passive commuters, 47.7% lived within 3.0 kilometers from school.

**Figure 2:**
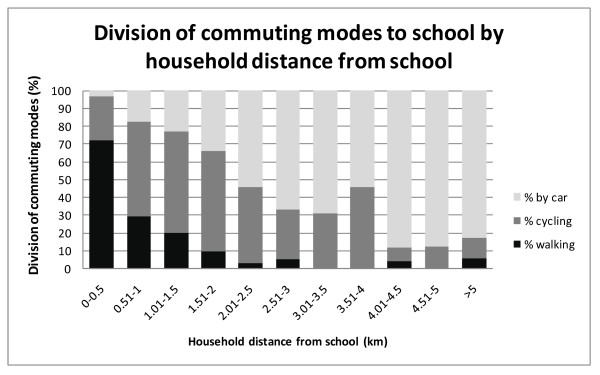
**The division of commuting modes to school by household distance from school**.

Figure 2 shows the division of commuting modes by household distance from school. The percentage of children commuting by car increases, while the number of children using active commuting modes decreases when the household distance from school increases.

**Figure 3:**
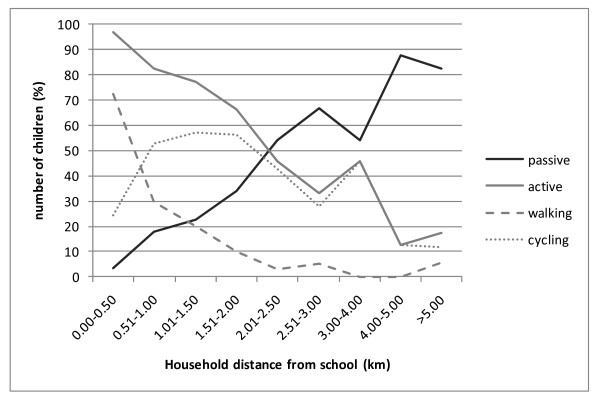
**Percentages of children going to school by active or passive commuting per distance range**.

Figure 3 represents the number of children (%) that walked, cycled or commuted passively to school per distance range. In the sample of the active commuters, the number of cyclists exceeded the number of children who walked to school in the range from 0.51 - 1.00 kilometers. In the range of 2.01-2.50 kilometers, the number of passive commuters exceeded the number of the active commuters. There is a decrease in the number of children using motorized transport to go to school and an increase of active commuting, due to an increase in the number of children cycling to school in the range of 3.00 - 4.00 kilometers. This can be due to the smaller sample size (n = 53) in this distance range. We would expect a continuous increase in the number of passive commuters, and a continuous decrease in the number of active commuters as the household distance from school increased.

### Correlates of active commuting

Correlates of active commuting were determined for children living within a distance of 3.0 kilometers from school (n = 503). This distance was set as the criterion distance for cycling to school in elementary school children. Within this distance range (0.00 - 3.0 kilometers), children whose parents reported a greater accessibility to walk (OR = 1.83; 95% CI = 1.38-2.44) were more likely to commute actively to school. The perceived walkability index, walking/cycling facilities, neighborhood aesthetics, safety from traffic and crime and the number of cars in a household family; were all not significantly associated with transport mode choice for children living within a distance of 3.0 kilometers from school.

Within the group of all active commuters (n = 369) living within the criterion distance of 3.0 km, only the household distance from school was significantly associated with commuting to school by bike instead of going to school on foot (OR = 7.24; 95% CI = 2.56-20.51). Active commuting children living further away from school, but still within the criterion distance of 3.0 kilometers, will prefer to commute to school by bike instead of going on foot. After correction for household distance from school, no other environmental correlates were found.

## Discussion

The prevalence of active commuting in the present sample of 696, 11- to 12-year-old children in Belgium, (59.3%) is much higher than the prevalence found in Australia [17], the USA [23], and European countries (e.g. Scotland, France, Portugal,..) [29]. As Flanders in Belgian has a mild sea climate, a flat landscape and a dense network of cycle paths; rates of active commuting to school can be much higher compared to other non-cycle-friendly regions. Another explanation can be that in Belgium, children from elementary schools live relatively close to their school (mean distance to school 2.96 ± 3.97 kilometers) compared to children in Australia and the USA [41], as the distance to school is the most important negative predictor of active commuting. Results from this study showed that the further children lived from school, the less frequently they actively commuted to school by walking or cycling. This finding is consistent among our study and studies conducted in the USA [41] and Australia [16,17].

As the distance to school increases, active commuting children go more to school by bike instead of going to school on foot. The criterion distance for walking (1.5 kilometers) is comparable with the criterion distance reported in older adolescents by Van Dyck et al. [18]. The criterion distance for cycling, on the other hand, is much higher in older adolescents (8.0 kilometers) than the distance of 3.0 kilometers found in this study for children. As cycling is a complex task and requires more attention than walking [42], younger children may be limited in their independent mobility by their parents to ensure the safety of their child. Although, the results of this study revealed that 3.0 kilometers is a feasible distance for 11- to 12-year-old children for active commuting to school, 47.7% of the passive commuters lived within the criterion distance for cycling to school. Therefore, future interventions that are promoting active commuting, need to focus on this group of passive commuters in which almost half of the passive commuters could be reached. As it is not possible to modify household distance from school, a different approach will be needed to encourage children to commute actively to school when they are living further than 3 km away from school. A possible way is to create 'drop off spots' at 1.5 km from school (the criterion distance for walking), where parents can drop their children e.g. on their way to work. Teachers or volunteers can guide the children from the drop spots to school and from school to the drop spot to guarantee the safety of the children. Also other safety issues need to be taken into account: e.g. children should be advised to wear a fluorescent traffic safety vest. Kingham et al. (2007) showed in elementary schoolchildren that supervised walking pools are feasible and have many advantages such as social, timesaving, safety and health benefits. Moreover, children get used to walking and this can increase walking behavior in other family members [43].

In the range of 2.01 - 2.50 kilometers distance to school, the number of passive commuters already exceeded the number of active commuters. This is mainly due to a smaller number of children who walk to school and a larger number of children who are driven to school from a household distance of more than 2.0 kilometers from school. From a household distance of 2.0 kilometers from school, less than 4% of the children went to school on foot, 23% went to school by bike and 73% went to school by car.

By determining correlates of active commuting to school for children who are living within the criterion distance for cycling (3.0 kilometers), only accessibility to walk seemed to be positively associated with active commuting to school. A better accessibility to walk includes an easy way to walk to school and many places where children can easily walk to. Furthermore, in neighborhoods with a better accessibility, there are many places where children can walk to alone or with someone else and it is easy to walk to a play ground or a park.

It is mainly the responsibility of the government to improve the accessibility. Improving accessibility brings along radical changes. This includes the improvement of the number and condition of the cycle paths and tracks. However, it may include other features, as the definition of accessibility is unclear in the NEWS-Y questionnaire. Improving the accessibility will require time and money and is a challenge for urban planners. The implication of improving accessibility needs further research, especially the cost-effectiveness of such interventions needs to be studied, as these studies are rather scarce, especially in children, where no studies were found. Two studies investigating cost-effectiveness showed that community- and street-scale urban design and land use policies and practices can be effective in enhancing physical activity levels in adults [44,45].

Besides "improving accessibility" it will be necessary to pay attention to other potential (non-environmental) influencing factors. It would not be sufficient to only modify the environment. On the other side, focusing on individual behaviors only, when the environment is not supportive, will produce weak and short term effects [46]. According to Sallis et al., the most effective interventions are those that focus on four domains: intrapersonal, social, physical environmental, and policy [13].

Within the criterion distance of 3.0 kilometers, the further the children lived from school, the less they went to school in an active way. Even within the criterion distance range, distance is an important correlate of active commuting to school. It is rather surprising that safety from traffic and crime, walking and cycling facilities, and walkability were not associated with active commuting to school, as shown in other studies [16,21-23]. A possible explanation may be the fact that during the last years, the government in Belgium has invested in improving the safety around schools. Crossing guards in front of the school on crosswalks, traffic lights, speed limits of 30 km/h around schools are all implemented in most Belgian elementary schools and walking and cycling facilities around schools are mostly in good condition.

As found in Merom et al., the number of cars per household was not associated with the choice of transport mode to school [17]. As only 2.0% of the households in this study did not have a car or another motorized vehicle, a lack of power can be a possible explanation. Probably the influence of having no car versus having one or more cars will mainly make the biggest difference on active commuting mode choice to school in children.

Among the active commuting children, bicycle use in children was positively associated with a longer household distance from school. The main explanation for this finding is probably the fact that it is usually faster to bike than to go on foot. None of the perceived environmental correlates by the parents did determine if active commuters went to school on foot or by bike.

A first strength of the study was the sample size (n = 696). Compared by other similar studies, the present sample size is relatively large; as it exceeds most sample sizes of similar studies. The second strength was the use of parental perceptions instead of children's perception, as the parents are usually the main decision makers for the transport mode choice to school from their children. Thirdly, the use of the NEWS-Y questionnaire, a validated questionnaire, is also a strength of this study. Finally, distance to school was objectively determined using online Routenet route planner.

This strength also entails a weakness, as over- or underestimation of the distance is possible when using an online routeplanner. The active traveled way can be shorter (if short cuts exist) or longer (if longer but safer routes exist) than the distance calculated with the online route planner.

Another limitation is the fact that some children can be driven to school in the morning but going home on foot in the afternoon or the other way around. Furthermore, the questionnaire did not make a distinction between active, passive or 'mixed transport' commuters.

According to the results, there are children who walk to school and live further than 5 km away from school. This might be due to combined modes of transportation. For example, it can be possible that children are dropped by their parents at their grandparents' place and that children continue their way to school on foot from that place. As combined modes of transportation were not included in the questionnaire, this was a limitation of this study.

In addition, youth attitudes, external factors, and parental attitudes were not included in the study as potential correlates of active commuting to school. However, these determinants may be more amenable in the short term than "improving accessibility". Further research in this domain is necessary. A last limitation is the cross-sectional character of the study, therefore, no causal relationships can be concluded. If commuting decreases over time in a neighborhood with sustained accessibility, the determinants will be more likely individuals factors. Longitudinal studies are necessary to confirm these thoughts.

## Conclusions

Household distance from school is the most important predictor of active commuting to school. According to the present results, interventions to promote active commuting in 11- to 12-year-old children should be focusing on children living within a distance of 3.0 kilometers from school by improving the accessibility to walk on the way from children's home to school.

In children who live further away from school, alternative strategies should be applied to enhance the daily physical activity levels. Schools could be encouraged to determine places at 1.5 kilometers from school where parents can drop their children. This should be a feasible distance for children to walk to school from that place.

Suggestions for further research may be the investigation of correlates of active commuting to school, based on objective observations of the neighborhood. It is possible that parents from active commuters make a different interpretation of the neighborhood as they are more aware of risks, and facilities along the way to school compared to parents from passive commuters. It might be interesting to compare the results based on subjective and objective neighborhood characteristics. The physical environment is one of the four main domains that can influence active travel behavior [15]. As there is only one perceived environmental factor associated with active commuting to school, it may be necessary to focus in interventions on other affecting factors such as individual factors and external factors [15].

By changing these individual factors (parental and children's attitude, self-efficacy,...) the criterion distances could possibly be moved to a further household distance from school and have an impact on numerous children, living further away than the 3.0 km the criterion distances. Further research may be necessary to develop appropriate interventions. Longitudinal studies are necessary to investigate causal relationships.

## Competing interests

The authors declare that they have no competing interests.

## Authors' contributions

SDH conducted the statistical analyses and drafted the manuscript. FDM developed the data collection protocol and coordinated the data collection. GC, IDB, BD and FDM participated in the interpretation of the data, helped to draft the manuscript and revised the manuscript for important intellectual content. All authors read and approved the final manuscript.
